# Structure and Zeatin Binding of the Peach Allergen *Pru p 1*

**DOI:** 10.1021/acs.jafc.1c01876

**Published:** 2021-07-14

**Authors:** Reiner Eidelpes, Florian Hofer, Manuel Röck, Sebastian Führer, Anna Sophia Kamenik, Klaus R. Liedl, Martin Tollinger

**Affiliations:** † Institute of Organic Chemistry, Center for Molecular Biosciences Innsbruck (CMBI), University of Innsbruck, Innrain 80/82, A-6020 Innsbruck, Austria; ‡ Institute of General, Inorganic and Theoretical Chemistry, Center for Molecular Biosciences Innsbruck (CMBI), University of Innsbruck, Innrain 80/82, A-6020 Innsbruck, Austria

**Keywords:** NMR structure, allergen, ribonuclease, cytokinin

## Abstract

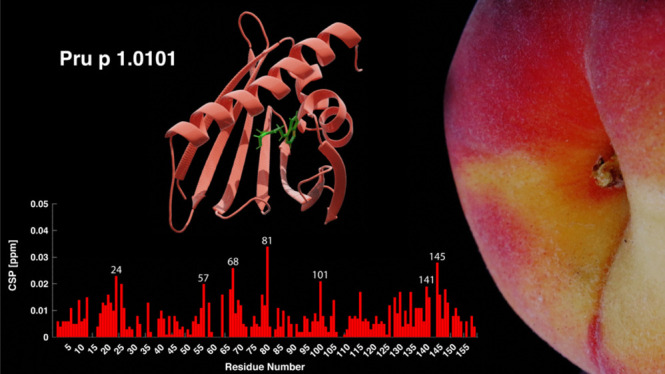

Peach (*Prunus persica*) is among the fruits most frequently reported to cause food allergies. Allergic reactions commonly result from previous sensitization to the birch pollen allergen Bet v 1, followed by immunological cross-reactivity of IgE antibodies to structurally related proteins in peach. In this study, we present the three-dimensional NMR solution structure of the cross-reactive peach allergen *Pru p 1* (isoform Pru p 1.0101). This 17.5 kDa protein adopts the canonical Bet v 1 fold, composed of a seven-stranded β-sheet and three α-helices enclosing an internal cavity. In *Pru p 1*, the inner surface of the cavity contains an array of hydroxyl-bearing amino acids surrounded by a hydrophobic patch, constituting a docking site for amphiphilic molecules. NMR-guided docking of the cytokinin molecule zeatin to the internal cavity of *Pru p 1* provides a structure-based rationale for the effect that zeatin binding has on the protein’s RNase activity.

## Introduction

Consumption of peach (*Prunus persica*) can elicit allergic reactions in atopic patients.^1,2^ Peach-allergic patients avoid eating these fruits, abstaining themselves from a valuable food source of high nutritive and health value. In Central and Northern Europe, peach allergy is strongly associated with birch pollinosis, resulting from initial sensitization to the major birch (*Betula verrucosa*) pollen allergen Bet v 1, followed by immunologic cross-reaction of IgE antibodies against Bet v 1 with structurally related proteins in peach.^3^ As a consequence, around 50% of all individuals suffering from birch pollinosis experience allergic reactions when consuming peach fruits or juice. Clinical symptoms are typically mild and include itching and swelling of lips, tongue, and throat but can progress to severe reactions and even anaphylactic shocks in some cases.^4^

The allergen that triggers birch-pollen-related food allergies (BPRFA) in peach is the 17.5 kDa protein *Pru p 1*.^5^*Pru p 1*, like Bet v 1, belongs to the class 10 of pathogenesis-related (PR) proteins that are activated in plants in response to abiotic and biotic stress.^6^ PR-10 proteins possess a canonical fold consisting of a seven-stranded β-sheet wrapped around a long α-helix at the C-terminus. Together with two additional short helices, these elements form an internal cavity, which binds numerous amphiphilic ligand molecules in vitro, including flavonoids, fatty acids, and plant steroids. For *Pru p 1*, which is encoded by a multigene family,^7^ three isoallergens with IgE reactivity have been identified, with sequence identities between them ranging from 74.8 to 78.0%. These proteins are present in both pulp and peel of peach fruits, with expression levels depending on the maturation stage and on the cultivar, ranging from 0.01 to 0.18 μg per gram of fruit, and maximum expression in the pit hardening phase.^8,9^ In addition, it could be shown that fruit bagging during growth, which is widely practiced in peach cultivation to improve fruit color and quality, significantly reduces the expression levels of *Pru p 1* genes.^10^

While structural data for *Pru p 1* allergens are not available to date, the immunological properties of these proteins have been studied in detail.^11^ Using ELISA and ImmunoCAP experiments, IgE antibody binding to recombinantly produced *Pru p 1* was demonstrated and immunologic cross-reactivity was shown. Notably, *Pru p 1* possesses ribonuclease (RNase) activity. Two independent studies established that isoallergens from the peach fruit (Pru p 1.0101, Pru p 1.0201) and peach pollen (Pru p 1.0301) efficiently hydrolyze RNA in vitro.^11,12^ RNase activity appears to be a common property of some, but not all, PR-10 proteins, including the food allergens from peach and strawberry,^6,13^ and represents a common defense mechanism of plants in response to pathogen infection.^14^ The mechanistic details of how PR-10 proteins catalyze the hydrolysis of RNA are unknown. It was observed, however, that the RNase activity of *Pru p 1* is modulated by the plant hormone zeatin, a cytokinin present in peach, suggesting a regulatory role of zeatin for RNase activity and stress defense.^11,12^ A possible functional link between the RNAse activity of *Pru p 1* and its capability to interact with zeatin is yet to be established.

In this study, we present the three-dimensional NMR solution structure of the peach allergen Pru p 1.0101, which shares 59.4% sequence identity with the sensitizing allergen from birch pollen, Bet v 1, along with an in-depth binding study of zeatin. For this particular isoallergen of *Pru p 1*, zeatin significantly decreases the rate at which RNA hydrolysis occurs. Knowledge of the three-dimensional structure of *Pru p 1* and its ligand binding characteristics are key for understanding the observed immunologic cross-reactivity of this allergen and for understanding examining its RNase activity on a molecular level.

## Materials and Methods

### Recombinant Protein Expression and Purification

The protocol for construction of plasmids encoding for *Pru p 1* isoallergens, recombinant expression, and purification has been described.^15^

### NMR Spectroscopy

NMR experiments were recorded at 25 °C on a 500 MHz Agilent Direct Drive 2 spectrometer equipped with a room temperature probe, using 0.5 mM ^15^N- and ^15^N/^13^C-labeled Pru p 1 samples in 10 mM sodium phosphate buffer pH 6.9, supplemented with 10% D_2_O. All experiments were recorded at 298 K. The existing backbone chemical shift assignment of Pru p 1.0101^15^ was complemented by ^15^N- and ^13^C-edited three-dimensional ^1^H-^15^N-NOESY-HSQC, ^1^H-^13^C-NOESY-HSQC, and aromatic ^1^H-^13^C-NOESY-HSQC experiments, each recorded with 150 ms mixing time. The NMR data were processed with NMRPipe.^16^ Backbone resonance assignments of Pru p 1.0201 and Pru p 1.0301 were obtained using standard triple-resonance methods as described.^15,17^ The CcpNMR software package was used for visualization and interpretation of NMR spectra.^18^

### Structure Calculation

Nuclear Overhauser effects (NOEs) were assigned and classified into four subclasses according to their peak intensities and were used as distance restraints. The upper restraint distance boundaries were 6 Å (weak), 5 Å (light), 4 Å (medium), and 2.8 Å (strong), and the lower boundary was set to zero. Dihedral angle restraints (Φ and Ψ) were predicted using the program TALOS+.^19^ Structure calculation employing XPLOR-NIH (version 2.52) followed the simulated annealing protocol, determining a set of 100 initial structures in 3000 steps at a temperature of 7000 K, followed by 10,000 cooling steps.^20^ The 20 lowest energy structures were further refined in an explicit solvent with the AMBER 19 simulation package using the AMBER ff14SB force field,^21^ where each structure was solvated in an orthogonal TIP3P water box, with a minimum wall distance of 10 Å. Each system was minimized, followed by a slow heating to 300 K and subsequent equilibration of the water box and the density. During all of these steps, the protein’s heavy atoms were kept fixed. Hereafter, each system was subjected to 10 sequential simulated annealing steps of 10 ns length each. During these steps, the protein atoms were restrained using the experimental NOE distance restraints, the violations were monitored, and a structural ensemble was generated. The quality of the final structures was validated using the protein structure validation software (PSVS) suite and in-house Python scripts.^22^

### Zeatin Binding

Titrations of *Pru p 1* with zeatin were performed by stepwise addition of the ligand (90 mM, dissolved in dimethylsulfoxide) to 450 μL samples of 0.2 mM ^15^N-labeled protein. Molar ratios were 1:16, 1:8, 1:4, 1:2, 1:1, and 1:0.5 Pru p 1/zeatin (≤3.5% (v/v) dimethylsulfoxide). A series of identical ^1^H-^15^N-HSQC experiments were recorded. In order to probe the interaction with zeatin, residue-specific chemical shift perturbations (CSPs) were determined as CSP = [((ΔδH)^2^ + (ΔδN/5)^2^)/2]^1/2^, where ΔδH and ΔδN are the ^1^H and ^15^N chemical shift differences between the apo protein and the complex, respectively.^23^ Seven amino acid residues with Δδ(^15^N) ≥ 0.15 ppm were selected for determining dissociation constants, *K*_d_, assuming binding of one equivalent of zeatin per *Pru p 1* molecule, as described.^24^

Two-dimensional ^1^H-^13^C heteronuclear NMR experiments were used for correlating phenylalanine and tyrosine side chain ^1^Hδ and ^1^Hε with side chain ^13^Cβ nuclei,^25^ with and without zeatin (8 mol equivalents) being present in the sample. For paramagnetic NMR experiments, N^6^-tetramethyl-1-piperidinyloxy (TEMPO)–adenine was synthesized from 6-chloropurine and 4-amino-2,2,6,6-tetramethylpiperidine-1-oxyl, as described in ref (26). Volumes of backbone amide resonances in ^1^H-^15^N-HSQC experiments (using an interscan delay of 5 s) in the presence of 1.7 mol equivalents N^6^-(TEMPO)–adenine before and after adding 12 mol equivalents of ascorbate in buffer were used for analysis. Translational diffusion of Pru p 1.0101 was probed using pulsed field gradient (PFG) diffusion NMR both in the absence and in the presence of zeatin as described,^27^ using a protein concentration of 0.2 mM and a zeatin concentration of 1.6 mM (1.75% (v/v) dimethylsulfoxide). Dioxane was used as a reference substance for the PFG-NMR diffusion measurements to determine hydrodynamic radii.^28^

### NMR Relaxation Measurements

^15^N relaxation dispersion (RD) experiments were recorded for the backbone amide resonances at 298 K on a 700 MHz Bruker AVANCE Neo spectrometer equipped with a Prodigy CryoProbe, as described.^29,30^ A sensitivity-enhanced Carr–Purcell–Meiboom–Gill (CPMG) pulse sequence with ^1^H continuous-wave decoupling during the CPMG period was used, employing CPMG field strengths of 33.3, 66.7, 100.0, 133.3, 166.7, 200.0, 266.7, 333.3, 446.7, 600.0, 733.3, and 933.3 Hz, with repeat experiments at 66.7 and 600.0 Hz and with the length of the CPMG pulse train set to *T*_relax_ = 30 ms. Partial peak volumes were obtained by adding the intensities in 5 × 5 grids centered on the peak maximum and converted to effective relaxation rates as *R*_2,eff_ = −1/*T*_relax_·ln(*I*/*I*_0_), where *I* is the partial peak volume at a given CPMG field strength and *I*_0_ is the partial peak volume in a reference experiment recorded with *T*_relax_ = 0. Fitting of the RD profiles was performed using in-house written scripts, as described.^31^

### In Silico Zeatin Docking

Docking of zeatin to Pru p 1.0101 was performed with the Molecular Operating Environment (MOE) version 2019.0102.^32^ The lowest energy structure of the refined NMR ensemble was used as the receptor. The protonate3d tool was employed to add hydrogens and assign protonation states according to a pH of 7.0.^33^ The ligand zeatin was set up within MOE, and docking was performed with the DOCK facility of MOE. Placement was done with the triangle matcher method and scored with the London Δ*G* scoring function. The top 200 scored poses were hereafter refined with the frozen receptor method and the GBVI/WSA Δ*G* scoring function. The best 20 poses were kept as final output. A representative pose was selected based on docking scores and NMR-measured CSPs.

## Results and Discussion

**Figure 1:**
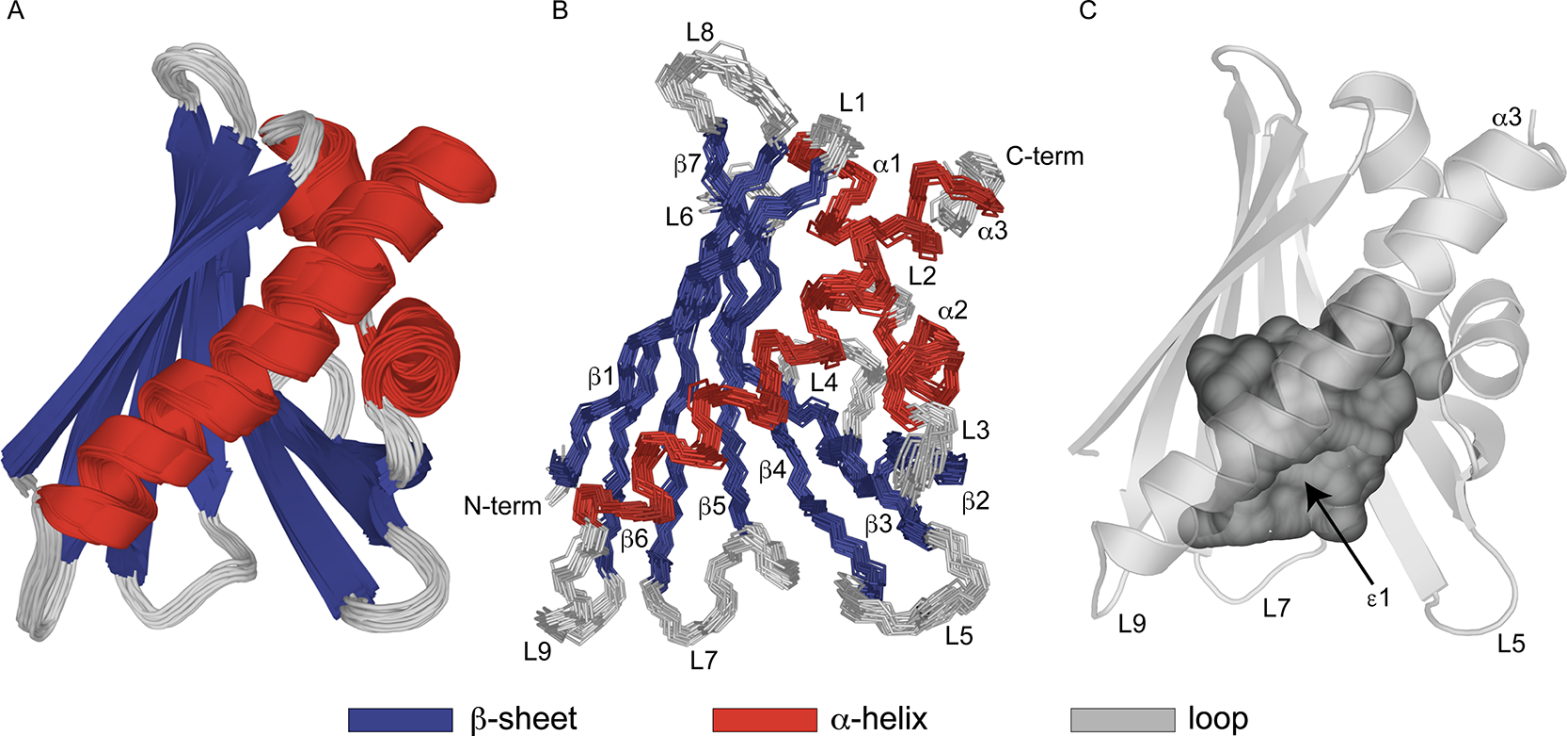
NMR solution structure of the peach allergen Pru p 1.0101 (pdb accession code 6Z98). (A) Ribbon representation of the ensemble of the 20 lowest energy structures. Secondary structure elements β1 (Val2-Ser11), β2 (Ile38-Glu45), β3 (Gly51-Phe58), β4 (Gly65-Asp75), β5 (His79-Ile86), β6 (Leu95-Ala106), β7 (Ser112-Thr122), α1 (Pro15-Val23), α2 (Ala26-Ala34), and α3 (Glu130-His154) are colored in blue and red. (B) Backbone overlay of the 20 lowest energy structures. Secondary structure elements, loops (L1–L9) and N- and C-termini are labeled. (C) Internal cavity of Pru p 1.0101, which is accessible *via* the entrance ε1 formed by the N-terminal end of helix α3 and loops L5, L7, and L9. Figures were prepared using the program PYMOL.^52^

The three-dimensional structure of Pru p 1.0101 comprises a seven-stranded antiparallel β-sheet (β1−β7) and three α-helices, which adopt the canonical PR-10 fold (Figure 1). The two short consecutive helices α1 and α2 are located above the curved central β-sheet and act as V-shaped support for the long C-terminal helix α3. Together, these structural elements enclose a internal cavity that is accessible for ligands *via* an amphiphilic entrance, denoted ε1,^34^ which is delimited by the N-terminal end of helix α3 and loops L5 (connecting strands β3 and β4), L7 (between strands β5 and β6), and L9 (connecting β7 with α3). In total, the *Pru p 1* structure contains 43% β strand and 27% α helical structure, respectively, in good agreement with estimates from circular dichroism.^11,12^ The NMR structural ensemble of *Pru p 1* displays well-defined secondary structure elements and conformational homogeneity. The root-mean-square deviation (rmsd) values of the 20 lowest energy structures are 0.5 and 0.3 Å for heavy atoms and backbone atoms, respectively (Table 1).

**Table 1:** Input Restraints for the NMR Structure Determination and Refinement Statistics

Experimental Restraints	
total no. of NOE-based distance restraints	2982
intraresidue [*i* = *j*]	1035
sequential [|*i* – *j*| = 1]	706
medium range [1 < |*i* – *j*| < 5]	527
long range [|*i* – *j*| ≥ 5]	714
dihedral angle restraints	261
hydrogen bond restraints	107
total no. of restraints	3243
total no. of restraints per residue	20.4
long range restraints per residue	4.5
Restraint Violations	
average distance violation	0.00115 ± 0.00002 Å
max distance violation	0.1 Å
average dihedral violation	0.129 ± 0.024°
max dihedral angle violation	26.24°
rmsd Values (PSVS)	
backbone atoms	0.3 Å
heavy atoms	0.5 Å
bond lengths	0.014 Å
bond angles	2.6°
Ramachandran Plot Statistics (PSVS)	
most favored regions	93.6%
allowed regions	5.8%
disallowed regions	0.6%

**Figure 2:**
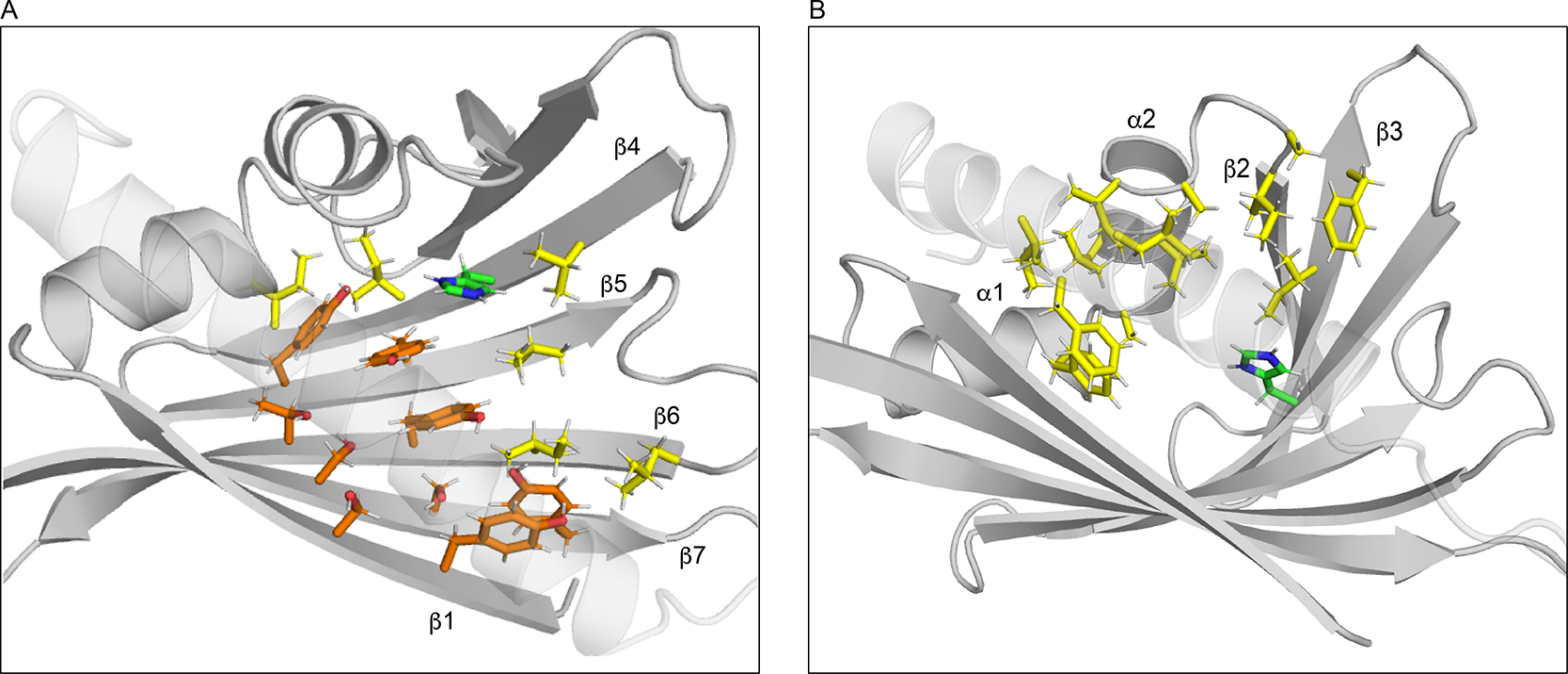
Internal cavity of Pru p 1.0101. (A) Strands β1 and β4−β7 contain nine amino acid residues (shown in orange), whose side chain hydroxyl groups are oriented toward the inner surface of the cavity, which are flanked by hydrophobic residues (yellow). The single histidine residue, His69, is shown in green. (B) Opposite face of the cavity is formed by hydrophobic residues in helices α1−α3, strands β2−β3 and loops L2 and L3. For illustrative purposes, the backbone of helix α3, which covers the internal cavity, is transparent in both panels.

The volume of the internal cavity of PR-10 proteins is largely determined by the geometry and the amino acid composition of the C-terminal helix α3 and displays considerable variability.^6^ For Pru p 1.0101, the internal cavity is relatively small (see Supporting Table 1), in part due to a slight inward kink of helix α3 and the presence of numerous bulky amino acids in this helix whose side chains point toward the protein interior (Leu143, Phe144, and Ile147). The inner surface of the cavity in Pru p 1.0101 is amphiphilic, formed by the side chains of hydrophobic aliphatic (Ile33, Ile38, Ile56, Ile71, Ile98, Ile147, Leu85, Leu95, Leu143, Val30, Val67, Ala26, Ala34, Ala37, and Ala140) and aromatic (Phe22, Phe58, and Phe144) residues, along with a number of hydroxyl-bearing amino acids (Tyr5, Ser7, Tyr81, Tyr83, Tyr100, Thr102, Ser116, Ser118, and Tyr120) and a histidine (His69). Intriguingly, all hydroxyl-bearing residues are located in the central β-sheet in the adjacent stands β1 and β4−β7, conferring a considerably polar character to the “bottom face” of the protein’s cavity (Figure 2A). The side chains of these residues offer an array of nine OH groups as anchoring possibilities for polar ligand molecules. The opposite face of the cavity, formed by helices α1−α3, strands β1 and β2, and loops L2 and L3, on the other hand, is hydrophobic (Figure 2B), underlining the spatial segregation of polar and hydrophobic surface residues in the cavity. This structural feature is fairly specific for *Pru p 1* since the nine hydroxyl-bearing residues in the β-sheet are not strictly conserved in other PR-10 plant food allergens (Supporting Information Figure S1). While, on average, almost 80% of these positions are indeed occupied by Tyr, Ser, or Thr in PR-10 allergens from*Rosaceae*, including strawberry Fra a 1 and apple Mal d 1, this figure drops to 50% and below in*Fabaceae* and*Apiaceae*. Of note, the single ionizable residue inside the cavity of *Pru p 1*, His69 in strand β4, is located at its inner end.

**Figure 3:**
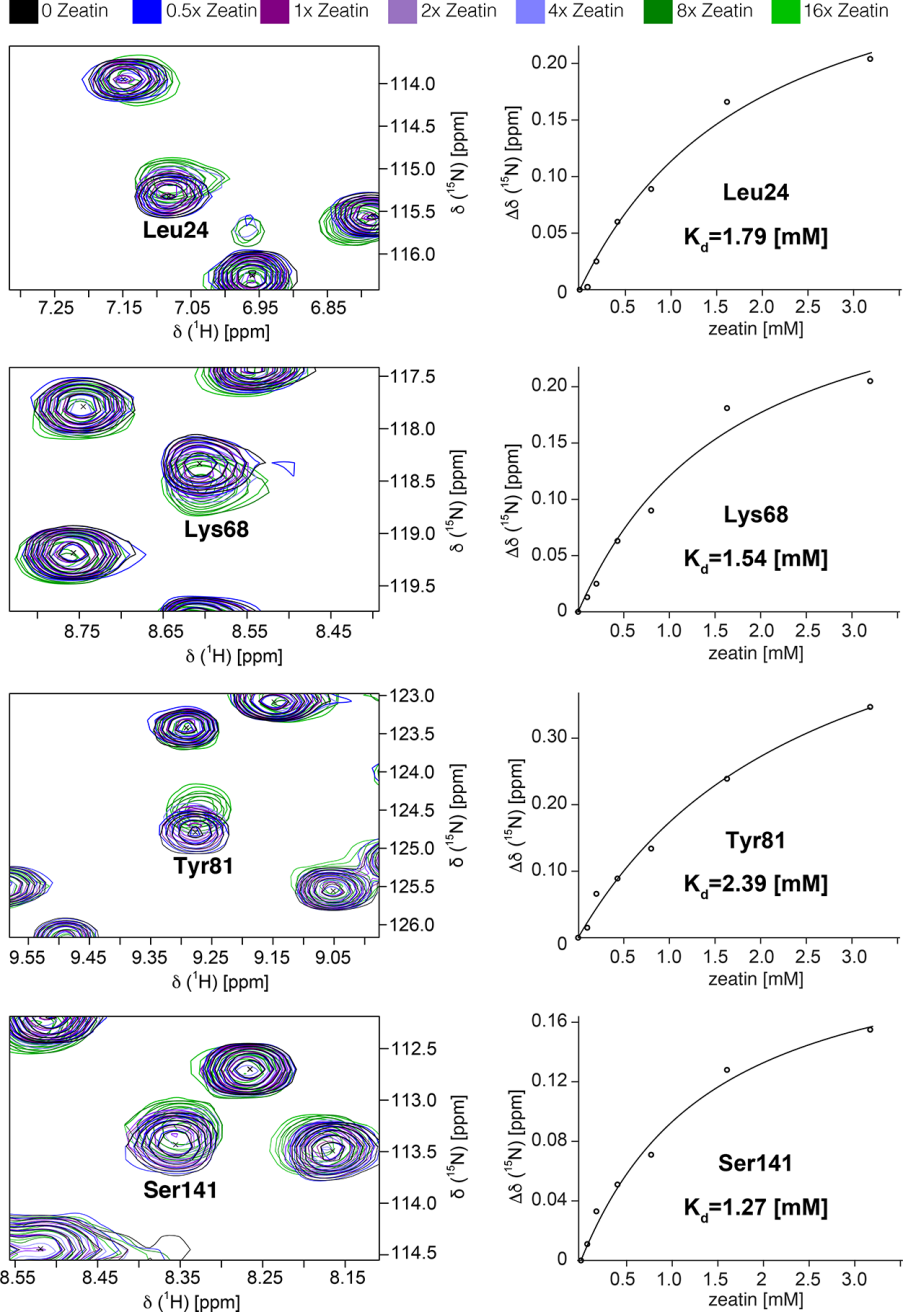
Binding of *trans*-zeatin to the peach allergen Pru p 1.0101. (A) Sections from backbone amide ^1^H–^15^N HSQC spectra of Pru p 1 (0.2 mM) in the presence of variable amounts (up to 16-fold excess) of zeatin, recorded at 500 MHz. Data of four representative amino acid residues (Leu24, Lys68, Tyr81, and Ser141) are shown. (B) Zeatin binding curves for four representative amino acid residues. *K*_d_ values for zeatin binding to Pru p 1.0101 were derived from ^15^N chemical shift changes, Δδ(^15^N), of these residues upon ligand binding by nonlinear least squares fitting of the binding curves as described.^24^ Additional experimental data for residues with Δδ(^15^N) exceeding 0.15 ppm are provided in Supporting Information Figure S2.

We used two-dimensional NMR titration experiments to study the interaction of the cytokinin zeatin to the peach allergen Pru p 1.0101 in solution. Addition of zeatin results in gradual chemical shift changes of backbone amide resonances of the protein, which is characteristic for intermediate to fast exchange on the NMR chemical shift timescale and typically observed for low affinity binding (Figure 3). Chemical shift changes, in particular, those of backbone amide ^1^H nuclei, in *Pru p 1* are small, indicating that zeatin binding is accompanied by only minor structural adaptions. Quantitative analysis of the ^15^N chemical shift data yields a complex dissociation constant *K*_d_ = 1.9 ± 0.4 mM. This value is 3 orders of magnitude higher than the *K*_d_ that was derived from isothermal titration calorimetry (ITC), which may be related to the different experimental conditions used.^12^ A similar disparity of *K*_d_ values obtained from different techniques has also been reported for zeatin binding to cytokinin-specific binding protein (CSBP), a PR-10 protein from*Vigna radiata*.^35^ To further probe zeatin interaction with Pru p 1.0101, we recorded RD NMR experiments in a 50% saturated sample. Several amino acid residues show increased RD rates (Supporting Information Figure S3), in accordance with rapid ligand binding and release and a mean residence time of bound zeatin in the millisecond range.^31^ This observation is in agreement with the common ability of PR-10 proteins to promiscuously bind low-molecular-weight compounds.^36^ The Pru p 1.0101 ITC data reported by Zubini et al.^12^ and our CSP data are both consistent with binding of one equivalent of zeatin per protein molecule.

**Figure 4:**
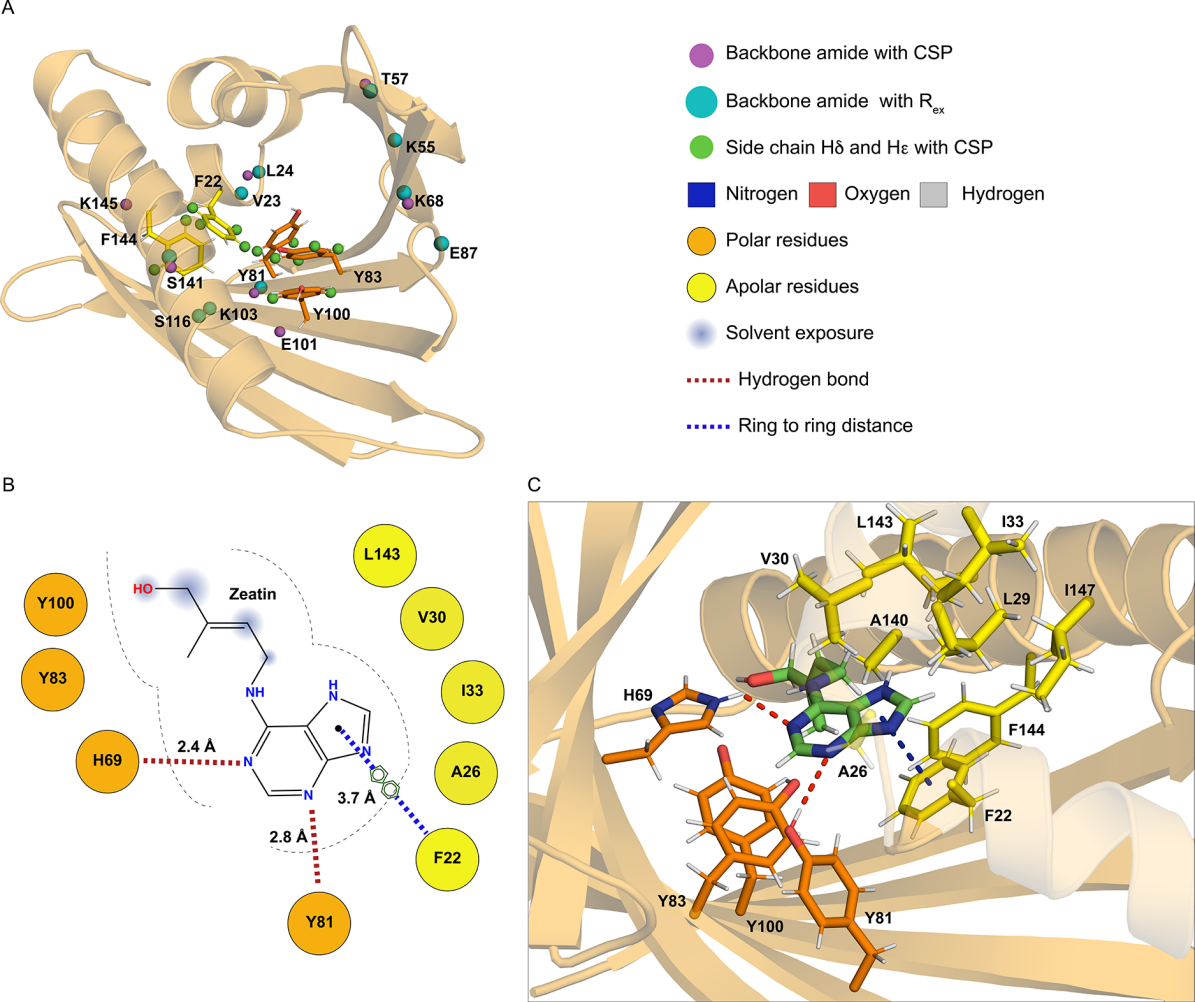
Interaction site of *trans*-zeatin. (A) Protein backbone of Pru p 1.0101 as viewed from the entrance to the internal cavity, ε1. Backbone amides with significant CSPs or increased ^15^N RD rates (*R*_ex_) upon zeatin binding are represented as purple and cyan spheres, respectively. Side chain ^1^Hδ and ^1^Hε nuclei of phenylalanines and tyrosines with CSP are displayed as green spheres. (B) Schematic and (C) detailed structural representation of the molecular docking model. Side chains of amino acids surrounding zeatin (distance ≤ 3.5 Å) are displayed in orange (tyrosine and histidine) and yellow (hydrophobic residues), while zeatin is shown in green. Red dashes represent hydrogen bonds from the imidazole side chain of His69 to N1 and from the side chain hydroxyl group of Tyr81 to N3 of the adenine moiety of zeatin. Blue dashes indicate the π-stacking interaction between the aromatic side chain of Phe22 and the purine ring system of zeatin. For illustrative purposes, in panel (C), helices α1 and α2 and Ala26 in loop L2 are transparent.

The CSP data and the RD data provide residue-specific information on the binding site of zeatin to *Pru p 1*. Figure 4 shows the amino acid residues, whose backbone amide ^15^N chemical shifts change measurably upon zeatin binding. They are located in the central β-sheet (Thr57, Lys68, Tyr81, and Glu101 in strands β3, β4, β5, and β6, respectively) at the bottom face of the cavity between helices α1 and α2 (Leu24) and in the central, hydrophobic part of helix α3 (Ser141 and Lys145), surrounding the internal cavity of the protein. The backbone amides that show increased RD rates in 50% saturated samples (amino acids Val23, Leu24, Lys55, Thr57, Lys68, Tyr81, Glu87, Lys103, Ser116, and Ser141) overlap with these residues or are in close proximity to them, in accordance with the notion that both experimental probes report on interactions with the ligand molecule.

In addition, we recorded side chain ^1^Hδ and ^1^Hε chemical shifts of aromatic residues (phenylalanine and tyrosine) in *Pru p 1* and their changes upon adding zeatin in ^1^H-^13^C heteronuclear correlation NMR experiments (Supporting Information Figure S4). As with backbone amides, only small CSPs are detectable and exclusively for residues whose side chains are oriented toward the internal cavity (Phe22, Tyr81, Tyr83, Tyr100, and Phe144). Their positions in the *Pru p 1* structure are in good agreement with the backbone amide CSP and RD data (Figure 4). Phenylalanines and tyrosines that are located on the protein surface (e.g., Tyr64, Tyr66, Tyr150, and Tyr158), however, are not measurably affected by zeatin, indicating that zeatin interacts with the protein’s interior.

Finally, we used paramagnetic relaxation NMR to probe binding of a synthetic analogue of zeatin to *Pru p 1*. For this purpose, a paramagnetic spin label, TEMPO, replacing the isoprenoid tail in zeatin, was attached to the exocyclic amine (N^6^) in adenine by chemical synthesis (Supporting Information Figure S5). Because paramagnetic spin labels cause faster relaxation of nearby nuclei, leading to reduced intensities, this molecule provides a sensitive tool for mapping its binding site. Indeed, addition of this paramagnetic zeatin analogue to *Pru p 1* reduces the intensities of numerous backbone amides, for some residues beyond detection, due to close spatial proximity to the TEMPO label. The data indicate binding of the ligand molecule to the protein’s internal cavity, as amino acids in the central β-sheet (β3 and β4) at the bottom face of the cavity, in helices α1 and α2, and at the inner side of helix α3 are the most affected by paramagnetic relaxation, while amino acids at the protein surface are only moderately perturbed.

In combination, the NMR chemical shift and relaxation data convey a coherent picture. The amino acid residues that are affected by zeatin, probed by either technique, cluster around the internal cavity in *Pru p 1*, including a number of phenylalanines and tyrosines (e.g., Phe22, Tyr81, Tyr83, and Tyr100), whose side chains form part of the internal surface. These aromatic residues are less than 8 Å (side chain-to-side chain) away from His69 at the inner end of the cavity but >12 Å away from those amino acids in loops L5, L7, and L9 and helix α3 (Phe58, Asp89, Ala90, Asn94, Ile128, His132, and Ala135) that form the amphiphilic entrance. This indicates that despite the relatively low affinity of zeatin and its rapid release, this cytokinin interacts with amino acid residues well inside the inner cavity of *Pru p 1*.

Using molecular docking, we derived a possible interaction site of zeatin that is consistent with the experimental data (Figure 4). According to this model, zeatin inside the cavity has direct contacts with hydroxyl-bearing residues in the central β-sheet. The adenine moiety of zeatin faces the inner end of the cavity and has hydrogen bond contacts to His69 and Tyr81 in strands β4 and β5, respectively, and π-stacks with the aromatic side chain of Phe22 in helix α1, which is distal from the lone opening of the cavity. In addition, the exocyclic amine (N^6^) of the adenine moiety is located in the vicinity of the side chain O–H of Tyr83 (predicted distance: 3.5 Å). The isoprenoid tail of zeatin at N^6^ is oriented toward the cavity’s entrance ε1, with the hydroxyl group at the tip of the tail in hydrogen bonding distance to side chain O–H of Tyr100 (predicted distance: 2.8 Å). Tyr81, Tyr83, and Tyr100 all form part of the hydroxyl-rich bottom face of the *Pru p 1* cavity and show small, but measurable, side chain CSPs upon zeatin binding. Of note, the isoprenoid tail of the bound zeatin molecule appears to be only loosely packed by hydrophobic residues (Val30 and Leu143) in the docking model, with the entrance to the cavity merely 5 Å (Phe58, Ala90) away from its hydroxyl group.

Our proposed interaction site in the internal cavity of Pru p 1.0101 is akin to a zeatin binding site in LIPR-10.2B from *Lupinus luteus*,^37^ with the adenine moiety interacting with hydrophobic residues in the short helix α1 and the isoprenoid tail being oriented toward the cavity entrance. Incidentally, LIPR-10.2B has an unusually large internal cavity, which accommodates up to three zeatin molecules in its interior (Table S1). For CSBP from*V. radiata*, which has a much smaller cavity volume, binding of one equivalent of zeatin per protein molecule was observed, deep inside the cavity, with a complex dissociation constant *K*_d_ ≈ 0.1 mM.^35^ Regarding Pru p 1.0101, binding of a single molecule of zeatin is in accordance with the relatively small size of its internal cavity, the NMR CSP data, and the reported ITC data.^12^ However, we cannot strictly exclude that a second zeatin molecule may bind to the protein. If a second ligand molecule indeed binds, it induces only minor chemical shift changes, indicating weak interactions and low affinity.

For the homologous birch pollen allergen Bet v 1, ligand binding causes compaction of the three-dimensional PR-10 scaffold.^28^ Likewise, catechin binding to the strawberry allergen Fra a 1 promotes the formation of a more compact structure, bending the central part of the helix α3 toward the bound ligand molecule and reducing the volume of the cavity.^38,39^ To explore the effect of zeatin binding to the compactness of the *Pru p 1* scaffold, we performed PFG translational diffusion NMR experiments with and without zeatin being bound. The data show that diffusion of *Pru p 1* is not measurably affected by the presence of zeatin. The hydrodynamic radius of the allergen is 18.4 Å, irrespective of whether zeatin is bound or not, in agreement with light scattering experiments reporting a hydrodynamic radius of 18 Å.^12^

**Figure 5:**
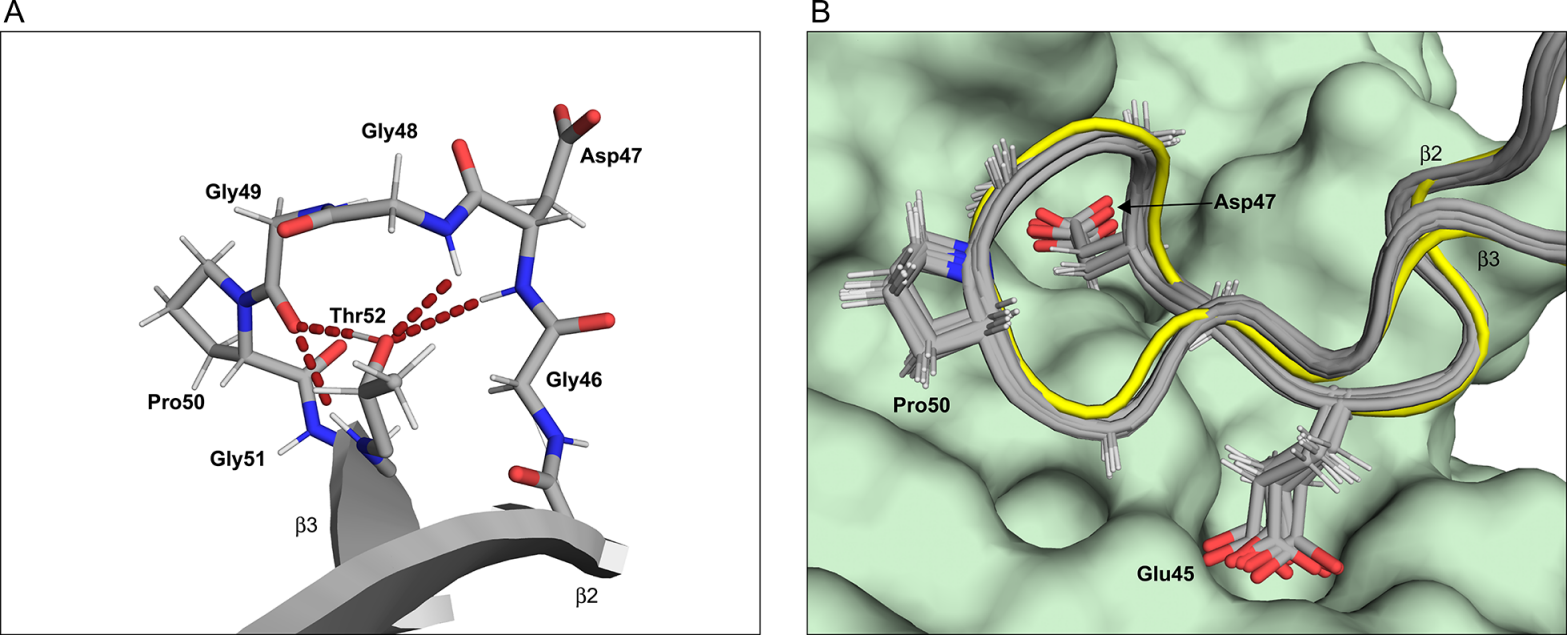
Structure of the β2–L4−β3 segment in Pru p 1.0101 encompassing the glycine-rich region, Gly46-Gly51. (A) Lowest energy structure of the glycine rich loop. Hydrogen bonds are indicated as red dashed lines. (B) Superposition of the β2–L4−β3 segment in *Pru p 1* (structural bundle, gray) with Bet v 1 (yellow) in complex with the antibody fragment Fab of BV16 (pale green, pdb entry code 1FSK). The side chains of *Pru p 1* residues between Glu45 and Gly51 are shown as sticks.

*Pru p 1* has been reported to hydrolyze RNA.^11,12^ The catalytic site and mechanistic details for the observed RNase activity of *Pru p 1* (and other PR-10 proteins) are not known to date. However, a functional role of the glycine-rich motif (Gly46-Asp47-Gly48-Gly49-Pro50-Gly51 in Pru p 1.0101) for RNA hydrolysis has been surmised due its high sequence similarity to the phosphate-binding loop (P-loop) of nucleotide-binding proteins.^40^ The glycine-rich motif was proposed to represent a possible nucleotide-binding site of PR-10 proteins by interacting with the phosphate group of RNA.^41^ In PR-10 proteins, this region is located in loop L4 and highly conserved. Our NMR solution structure of Pru p 1.0101 shows that hydrogen bonds between the backbone N–H groups of Asp47, Gly48, and the side chain oxygen of Thr52, as well as hydrogen bonds between the side chain O–H and the backbone N–H of Thr52 to the backbone carbonyl of Gly49 form a distinct network, which conveys structural stability to the glycine-rich region (Figure 5A). In nucleotide-binding proteins, however, the P-loop lacks the hydroxyl-bearing side chain of threonine (or serine) at position 52 and displays a different conformation, as noted previously for LIPR-10.1A and LIPR-10.1B from*L. luteus*.^42^ These structural data thus suggest that other elements are responsible for RNase activity.

Based on mutational studies, several amino acid residues have been reported as being important for the RNase activity of different PR-10 proteins.^43,44^ For ABR17, a PR-10 protein from*Pisum sativum*, mutation of histidine at position 69 to leucine drastically reduced RNase activity, suggesting that His69 is critical for its function.^43^ In *Pru p 1*, as in ABR17, His69 is located inside the cavity, in strand β4, with its polar and ionizable imidazole side chain oriented toward the inner surface (Figure 2). Histidine residues are often involved in RNA binding by acting both as proton donors as well as proton acceptors, and involvement in the observed RNase activity of PR-10 proteins is plausible.^45^ In our docking model, the imidazole side chain of His69 of *Pru p 1* hydrogen bonds with the purine base nitrogen atom N1 of the bound zeatin molecule, positioning the ligand close to the central β-sheet. Indeed, the neighboring amino acid in strand β4, residue 68, is among the two amino acids with the largest CSP upon zeatin binding (Supporting Information Figure S6), in agreement with the close proximity of the ligand molecule to strand β4. Interestingly, two independent studies reported that zeatin, when present in 20-fold excess, inhibits RNase activity of Pru p 1.0101.^11,12^ According to our structural model zeatin—by acting as weakly binding nucleoside analogue—might impede access of substrate RNA to His69 in the protein’s internal cavity. Histidine at this position is not conserved in PR-10 proteins, which may, in part, rationalize the observation that RNase activity is not an universal feature of these proteins. It was noted recently that tyrosine residues are generally conserved in PR-10 proteins that exhibit RNase activity.^13^ In *Pru p 1*, tyrosines are strongly represented in the immediate neighborhood of His69 and at the zeatin binding site, providing a range of anchoring possibilities for nucleosides.

Zeatin has been reported to modulate the RNase activity of *Pru p 1* in an isoallergen-dependent manner. Zeatin inhibits the RNase activity of Pru p 1.0101, while it has no effect on Pru p 1.0201 (for Pru p 1.0301, the results from the two available studies are contradictory).^11,12^ For Pru p 1.0201 and Pru p 1.0301, CSPs in titration experiments indicate that zeatin interacts at a similar location in the protein’s cavity as in Pru p 1.0101, with little effect on the three-dimensional structure of either isoallergen (Supporting Information Figure S6). In addition, the three isoallergens interact with zeatin with comparable affinities and *K*_d_ values between 0.6 and 1.9 mM. It remains unclear how exactly zeatin modulates the RNase activity of *Pru p 1* isoallergens in different ways.

The glycine-rich region is part of a conformational epitope that appears to be involved in the immunologic cross-reactivity between PR-10 proteins from birch pollen and various food sources.^38,46,47^ A central residue of the glycine-rich region, Glu45, has been identified as a critical structural feature in birch pollen Bet v 1, as mutation of Glu45 (Glu45 → Ser) abolished binding of the monoclonal IgG antibody BV16 to Bet v 1 and significantly reduced the IgE binding capacity of this protein.^48^ For the apple (Mal d 1) and the strawberry (Fra a 1) allergen, mutation of amino acids in the glycine-rich region measurably attenuated IgE binding,^38,47^ suggesting that this region is also part of an immunologically relevant epitope in these food allergens. Likewise, for the cherry allergen Pru av 1, significant reduction of IgE binding in blood sera was reported after mutation of Glu45.^46^ In accordance with their close phylogenetic relationship, cherry Pru av 1 and peach *Pru p 1* have highly similar amino acid sequences, with 100% sequence identity in the glycine-rich region.

In Bet v 1, the stretch between amino acids Glu42 and Thr52, which comprises the glycine-rich region, forms a contiguous surface patch that constitutes ca. 80% of the contact surface with the antibody BV16 (Figure 5B).^49^ Interactions between Bet v 1 and BV16 involve hydrophobic contacts and hydrogen bonding. Superposition of the glycine-rich region in Pru p 1.0101 and the complex of Bet v 1 with BV16 illustrates that the structure of this segment in the peach allergen is compatible with antibody binding. The side chains of the two polar residues Glu45 and Asp47 in *Pru p 1* are oriented in a fashion that is complementary to the surface of the antibody, with their carboxylates pointing into polar grooves in the antibody’s surface. In the Bet v 1-BV16 complex, the side chain of Glu45 forms hydrogen bonds with two backbone amides of the antibody, while Asp47 in *Pru p 1* is occupied by an asparagine (Asn47) in Bet v 1, which hydrogen-bonds to the side chain of a serine residue in BV16. As in the Bet v 1-BV16 complex, the conserved proline residue Pro50 is embedded in a shallow pocket on the surface of the antibody, accounting for a considerable portion of the contact surface between the molecules.

Comparison of pairwise rmsd values between Pru p 1.0101 and Bet v 1 in complex with BV16 shows that the conformation of the glycine-rich region is particularly conserved in these two proteins. The local rmsd of residues 45–52 is 0.3 Å (heavy atoms), while the total rmsd between the two structures is 0.8 Å. Addition of zeatin to *Pru p 1* does not produce structural changes of the glycine-rich region. Moreover, NMR RD experiments show that this segment is rigid and structurally homogeneous in solution (Supporting Information Figure S7), irrespective of whether the internal cavity is loaded with the ligand cargo or not. This is noteworthy in light of a recent study of the major hazelnut allergens Cor a 1.04, which revealed that antibody IgE binding and structural flexibility of these PR-10 proteins are correlated.^50^ Structural flexibility might represent a critical determinant that relates ligand binding of PR-10 allergens to immunologic reactions.^51^

## Supporting Information Available

Sequence alignment of PR-10 food allergens; supplemental figures containing NMR titration and RD data, and a comparison of cavity volumes (PDF)

## Abbreviations

PR-10: pathogenesis-related protein of class 10
CPMG: Carr–Purcell–Meiboom–Gill
CSBP: cytokinin-specific binding protein
ITC: isothermal titration calorimetry
NOE: nuclear Overhauser effect
PFG: pulsed field gradient
RD: relaxation dispersion
BPRFA: birch-pollen-related food allergy
