# Supplementary material for: Structure and Zeatin Binding of the Peach Allergen Pru p 1
Source: J Agric Food Chem. 2021 Jul 14;69(29):8120–9. doi: 10.1021/acs.jafc.1c01876 (PMC8323099; doi:10.1021/acs.jafc.1c01876)
Supplement: Supplementary file 1 — jf1c01876_si_001.pdf [file jf1c01876_si_001.pdf]

SUPPORTING INFORMATION

Structure and Zeatin Binding of the Peach Allergen Pru p 1

Reiner Eidelpes[1], Florian Hofer[2], Manuel Röck[1], Sebastian Führer[1], Anna Sophia Kamenik[2], Klaus R. Liedl[2], Martin Tollinger[1,*]

[1] Institute of Organic Chemistry, Center for Molecular Biosciences Innsbruck (CMBI), University of Innsbruck, Innrain 80/82, A-6020 Innsbruck, Austria

[2] Institute of General, Inorganic and Theoretical Chemistry, Center for Molecular Biosciences Innsbruck (CMBI), University of Innsbruck, Innrain 80/82, A-6020 Innsbruck, Austria

```
Pru p 1.0101     GVFTYESEFT SEIPPPRLFK AFVLDADNLV PKIAPQAIKH SEILEGDGGP GTIKKITFGE   60
Pru ar 1.0101    GVFTYETEFT SVIPPEKLFK AFILDADNLI PKVAPTAVKG TEILEGDGGV GTIKKVTFGE   60
Pru av 1.0101    GVFTYESEFT SEIPPPRLFK AFVLDADNLV PKIAPQAIKH SEILEGDGGP GTIKKITFGE   60
Mal d 1.0101     GVYTFENEFT SEIPPSRLFK AFVLDADNLI PKIAPQAIKQ AEILEGNGGP GTIKKITFGE   60
Pyr c 1.0101     GLYTFENEFT SEIPPPRLFK AFVLDADNLI PKIAPQAIKH AEILEGNGGP GTIKKITFGE   60
Fra a 1.0101     GVYTYENEFT SDIPAPKLFK AFVLDADNLI PKIAPQAVKC AEILEGDGGP GTIKKITFGE   60

Act c 8.0101     GVVTYDMEIP SKVPPVKLYK AFILDGDTLV PKVLPHAIKC VKILEGDGCA GTIKEVTFGE   60
Api g 1.0101     GVQTHVLELT SSVSAEKIFQ GFVIDVDTVL PKAAPGAYKS VEI-KGDGGP GTLKIITLPD   59
Dau c 1.0101     GAQSHSLEIT SSVSAEKIFS GIVLDVDTVI PKAAPGAYKS VEV-KGDGGA GTVRIITLPE   59
Gly m 4.0101     GVFTFEDEIN SPVAPATLYK ALVTDADNVI PKALD-SFKS VENVEGNGGP GTIKKITFLE   59
Ara h 8.0101     GVFTFEDEIT STVPPAKLYN AM-KDADSIT PKIID-DVKS VEIVEGNGGP GTIKKLTIVE   58
Cor a 1.0401     GVFCYEDEAT SVIPPARLFK SFVLDADNLI PKVAPQHFTS AENLEGNGGP GTIKKITFAE   60
                  *   .  *  * :    ::. .:  * *.:  **      .   :  :*:*   **:: :*: :


Pru p 1.0101     GSQYGYVKHK IDSIDKENHS YSYTLIEGDA LGDNLEKISY ETKLVASPSG -GSIIKSTSH   119
Pru ar 1.0101    GSQYAYVKHR VDGIDKDNLS YSYTLIEGDA LSDVIENIAY DIKLVASPDG -GSIVKTTSH   119
Pru av 1.0101    GSQYGYVKHK IDSIDKENYS YSYTLIEGDA LGDTLEKISY ETKLVASPSG -GSIIKSTSH   119
Mal d 1.0101     GSQYGYVKHR IDSIDEASYS YSYTLIEGDA LTDTIEKISY ETKLVACGSG --STIKSISH   118
Pyr c 1.0101     GSQYGYVKHR VDSIDEASYS YAYTLIEGDA LTDTIEKISY EAKLVASGSG --STIKSISH   118
Fra a 1.0101     GSHYGYVKHK IHSIDKENHT YSYSLIEGDA LSDNIEKIDY ETKLVSAPHG -GTVIKTTSK   119

Act c 8.0101     GSHHKCVKQR VDAIDKDNLT YSYTIIEGDV LAEKFESISY HIKIVACPDG -GSICKNRSI   119
Api g 1.0101     GGPITTMTLR IDGVNKEALT FDYSVIDGDI LLGFIESIEN HVVLVPTADG -GSICKTTAI   118
Dau c 1.0101     GSPITSMTVR TDAVNKEALT YDSTVIDGDI LLGFIESIET HLVVVPTADG -GSITKTTAI   118
Gly m 4.0101     DGETKFVLHK IESIDEANLG YSYSVVGGAA LPDTAEKITF DSKLVAGPNG -GSAGKLTVK   118
Ara h 8.0101     DGETKFILHK VESIDEANYA YNYSVVGGVA LPPTAEKITF ETKLVEGPNG -GSIGKLTLK   117
Cor a 1.0401     GNEFKYMKHK VEEIDHANFK YCYSIIEGGP LGHTLEKISY EIKMAAAPHG GGSILKITSK   120
                 ..    :  :  . ::.     :  ::: *   *    *.*   .  :.    *   :  *


Pru p 1.0101     YHTKGDVEIK EEHVKAGKEK ASNLFKLIET YLKGHPDAYN        159
Pru ar 1.0101    YHTKGDVEIK EEQVKAGKEK AAGLFKLVEA YLLANPDAYN        159
Pru av 1.0101    YHTKGNVEIK EEHVKAGKEK ASNLFKLIET YLKGHPDAYN        159
Mal d 1.0101     YHTKGNIEIK EEHVKVGKEK AHGLFKLIES YLKDHPDAYN        158
Pyr c 1.0101     YHTKGDIEIK EEHVKAGKEK AHGLFKLIES YLKDHPDAYN        158
Fra a 1.0101     YHTKGDVEIK EEHVKAGKEK ASHLFKLIEG YLKDHPSEYN        159

Act c 8.0101     YTTKGDCKVS EEEIKLGKEK AAEIFKALEA YLLANPDYC-        158
Api g 1.0101     FHTKGDAVVP EENIKYANEQ NTALFKALEA YLIAN-----        153
Dau c 1.0101     FHTKGDAVVP EENIKFADAQ NTALFKAIEA YLIAN-----        153
Gly m 4.0101     YETKGDAEPN QDELKTGKAK ADALFKAIEA YLLAHPDYN-        157
Ara h 8.0101     YHTKGDAKPD EEELKKGKAK GEGLFRAIEG YVLANPTQY-        156
Cor a 1.0401     YHTKGNASIN EEEIKAGKEK AAGLFKAVEA YLLAHPDAYC        160
                 : ***:     ::.:* .. :    :*: :*  *:  :
```

Supporting Figure 1. Multiple sequence alignment of PR-10 food allergens, obtained with Clustal Omega. The nine hydroxyl-bearing residues in the central β-sheet whose side chains contribute to the inner surface of the cavity in Pru p 1 are colored in red. *Rosaceae*: peach Pru p 1.0101, apricot Pru ar 1.0101, cherry Pru av 1.0101, apple Mal d 1.0101, pear Pyr c 1.0101, strawberry Fra a 1.0101; *Actinidiaceae*: golden kiwifruit Act c 8.0101; *Apiaceae*: carrot Dau c 1.0101, celery Api g 1.0101; *Fabaceae*: soybean Gly m 4.0101; peanut Ara h 8.0101; *Corylaceae*: hazelnut Cor a 1.0401. Conservation of amino acid residues is indicated by asterisks (identical), colons (conserved) and dots (semiconserved).

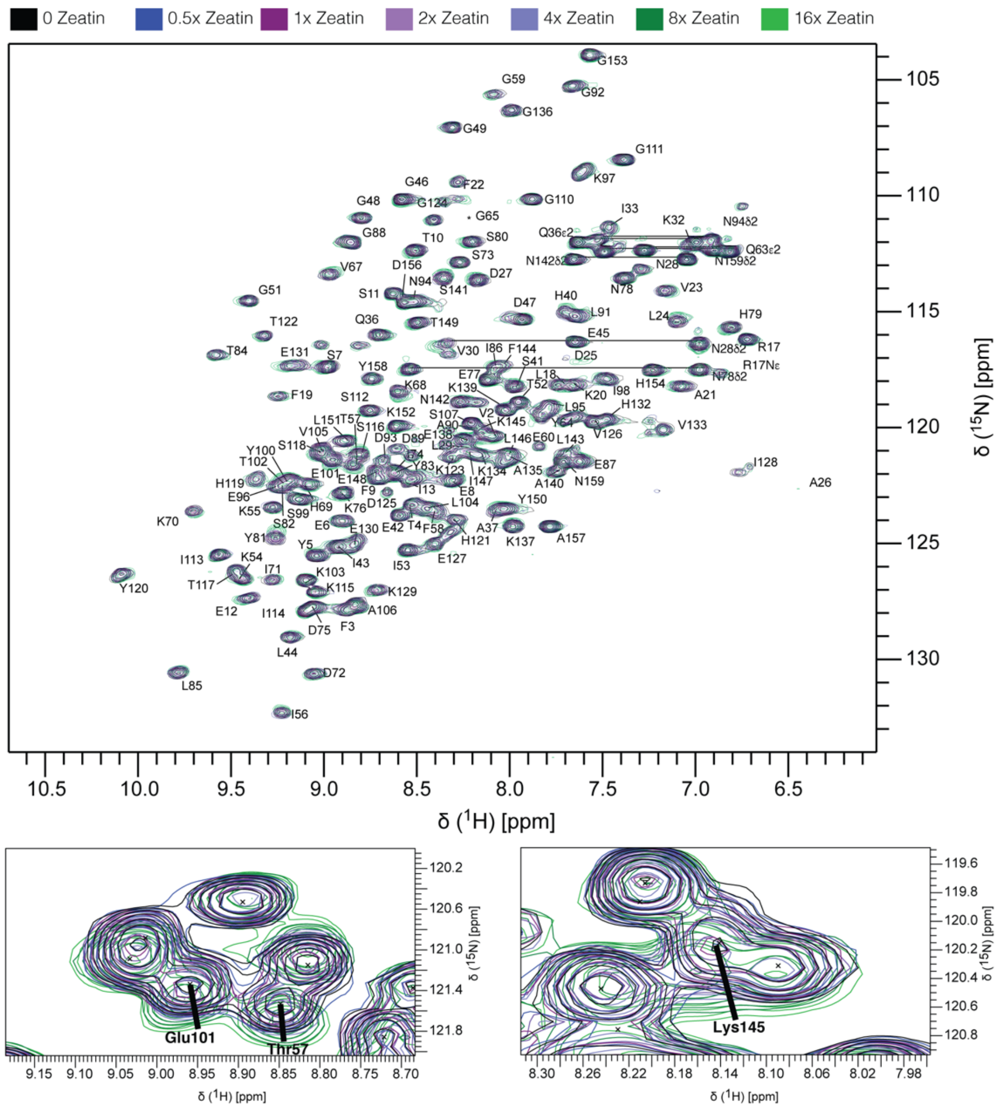

Supporting Figure 2. (A) ^{1}H-^{15}N HSQC spectra (500 MHz) of ^{15}N-labeled Pru p 1.0101 (0.2 mM) in the absence and presence of variable amounts (up to 16-fold excess) of unlabeled zeatin. (B) Complex dissociation K_d values for zeatin binding derived from the ^{15}N chemical shift titration data of residues Thr57, Glu101 and Lys145 were determined as described in the main text, yielding values of 2.36 mM, 1.60 mM and 2.54 mM, respectively.

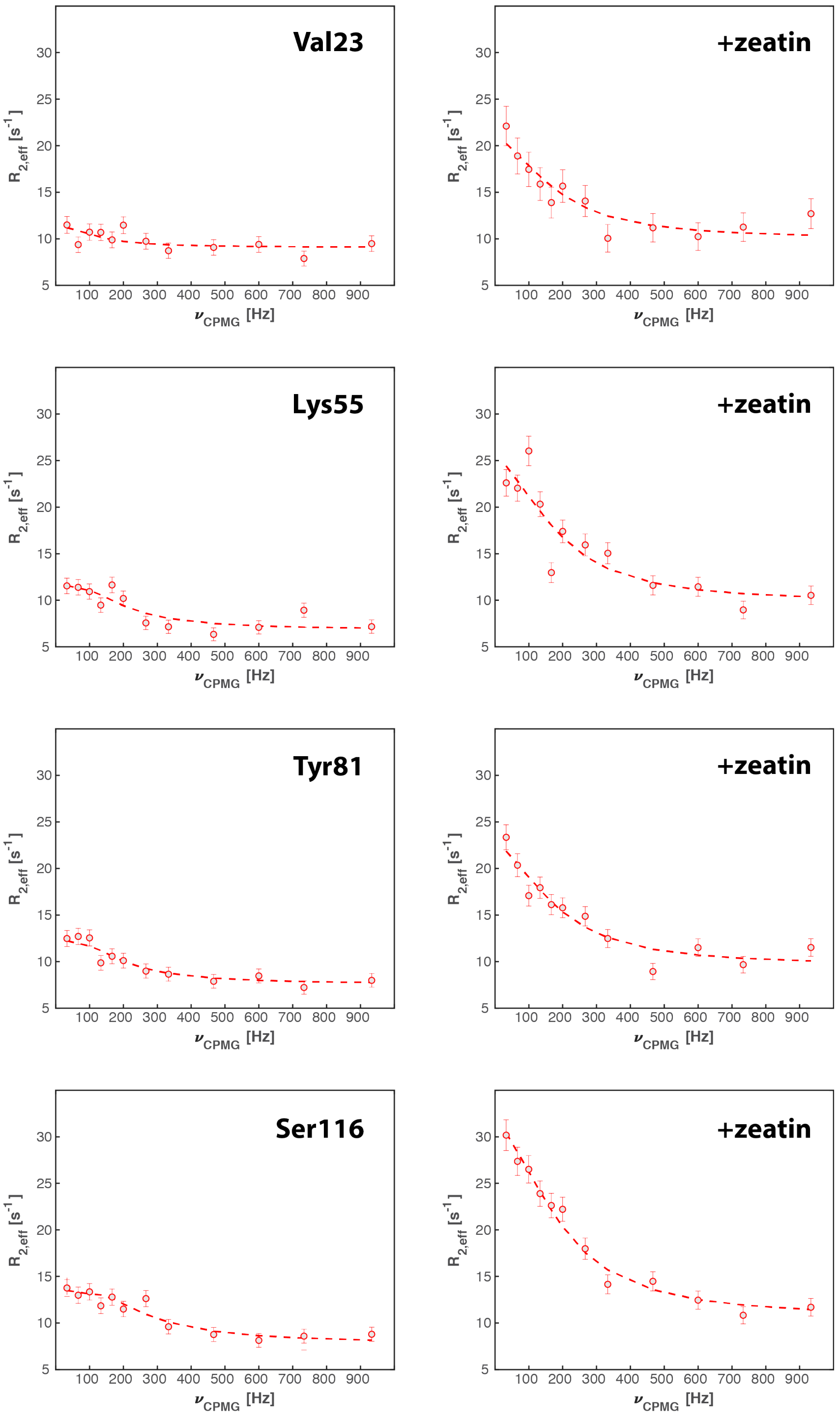

Supporting Figure 3. NMR relaxation dispersion (RD) data of Pru p 1.0101 before and after addition of zeatin (8-fold excess, ca. 50% saturation), recorded at 700 MHz. The backbone amide (^{15}N) RD profiles shown are representative for the zeatin binding site (Val23 in α1, Lys55, Tyr81 and Ser116 in the central β-sheet). Fits to the experimental data yield k_{ex} = 1100 ± 200 s^{-1} as exchange rate constant in the 50% saturated complex, corresponding to a mean residence time of the ligand in the millisecond range.

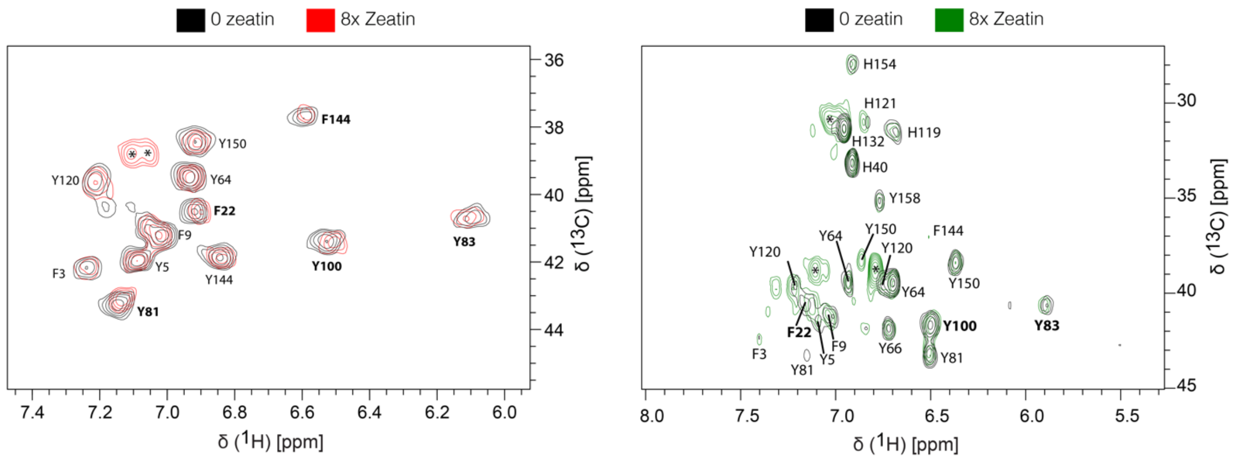

Supporting Figure 4. Two-dimensional ^{1}H-^{13}C NMR spectra showing correlations between side chain ^{1}Hδ (left) and ^{1}Hε (right) nuclei with ^{13}Cβ carbons of phenylalanine and tyrosine residues (500 MHz) in Pru p 1.0101 (0.72 mM), with and without of 8 mole equivalents of *trans*-zeatin being present. ^{1}H-^{13}C correlations with noteworthy chemical shift changes are marked in bold face. Residues that are not measurably affected by the addition of zeatin are labeled in dark gray. Asterisks indicate resonances for which assignments are not available.

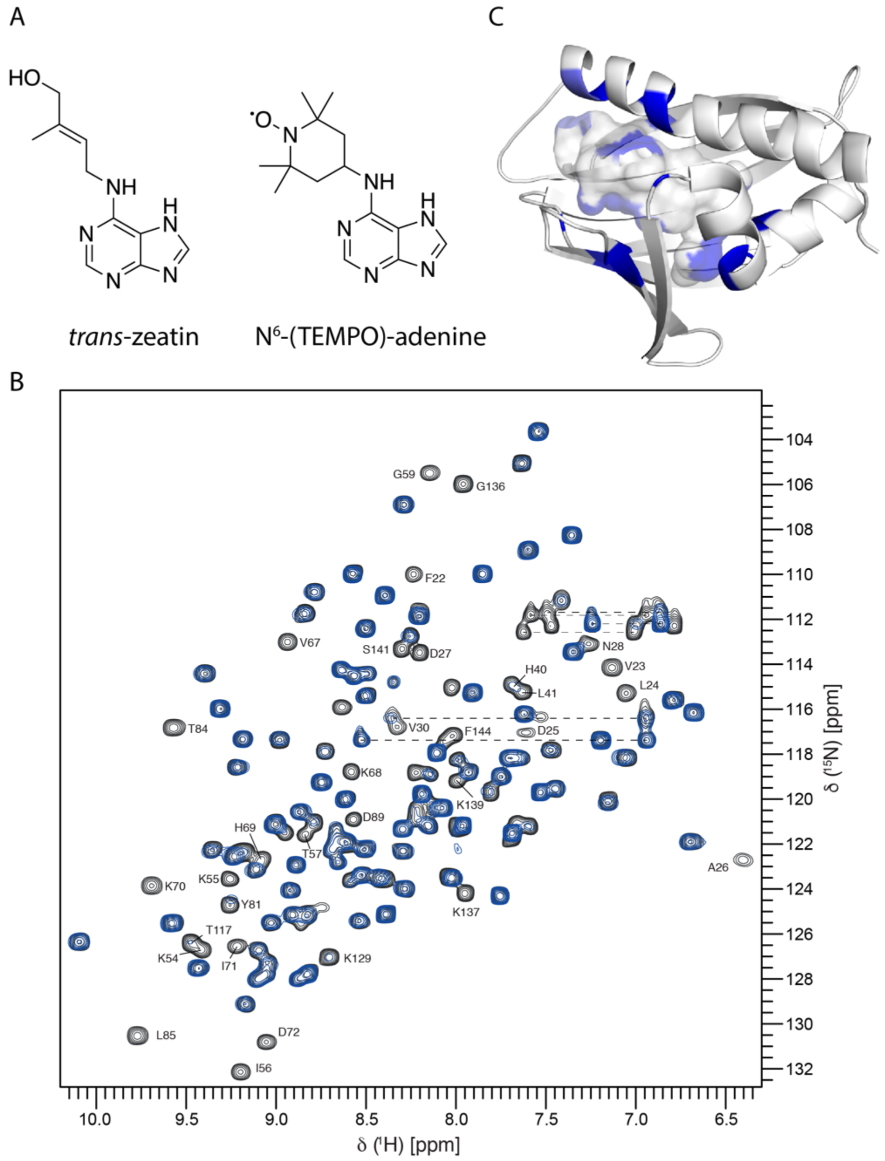

Supporting Figure 5. Paramagnetic NMR relaxation experiments. (A) Chemical structures of *trans*-zeatin and N⁶-(TEMPO)-adenine. (B) Backbone amide $^1H^{15}N$ HSQC spectra of Pru p 1.0101 (0.72 mM), with 1.7 mole equivalents of N⁶-(TEMPO)-adenine present, recorded at 500 MHz with an interscan delay of 5 s (blue). After addition of ascorbic acid to reduce the paramagnetic tag to its diamagnetic form, the spectrum shown in black is obtained. The resonances that are most affected by paramagnetic relaxation are labeled. (C) Backbone representation of Pru p 1.0101 highlighting residues that are affected by paramagnetic relaxation in blue, with darker color indicating larger effects.

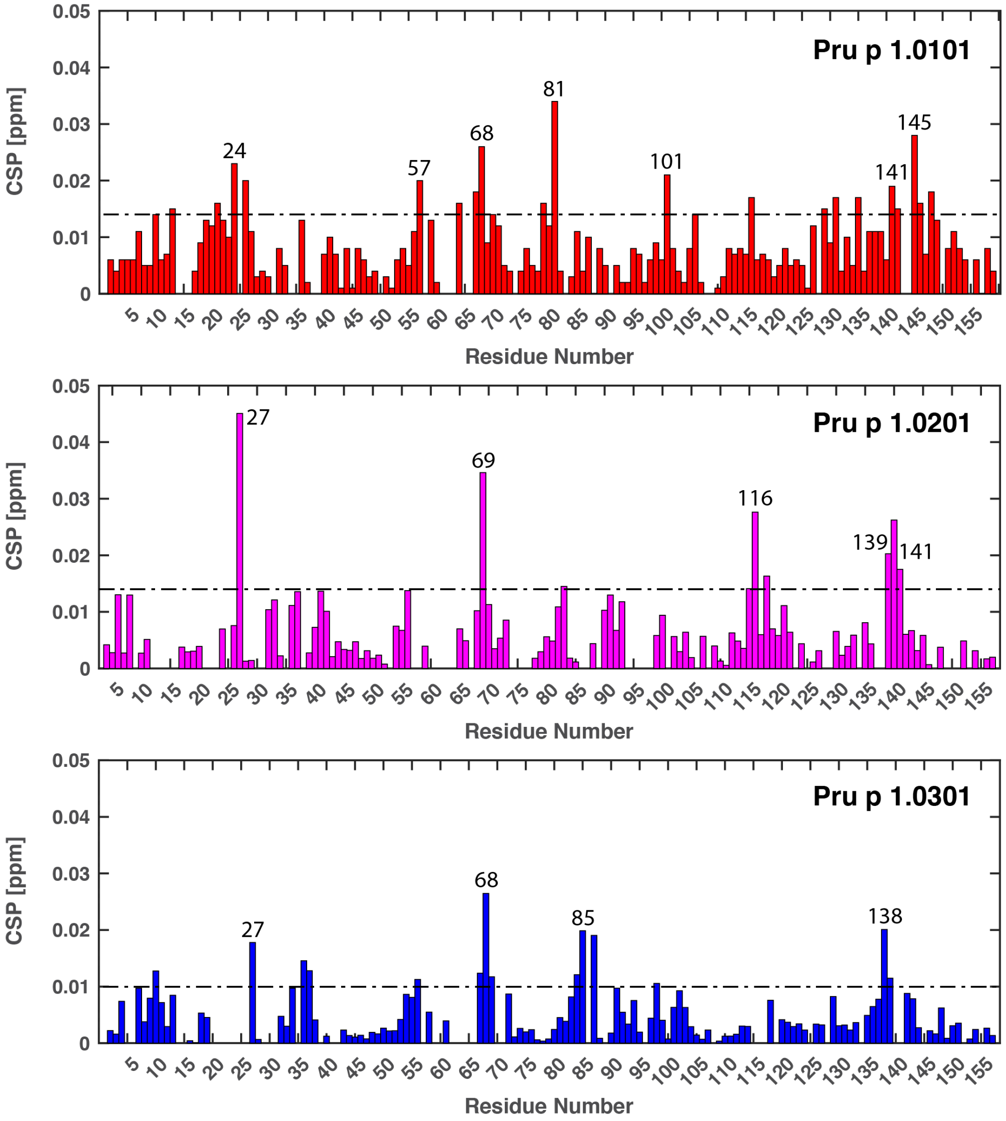

Supporting Figure 6. Residue-specific backbone amide chemical shift perturbations (CSP), determined as CSP = $[((\Delta\delta H)^2+(\Delta\delta N/5)^2)/2]^{1/2}$, where $\Delta\delta H$ and $\Delta\delta N$ are the ^{1}H and ^{15}N chemical shift differences between the apo protein and the complex, for the three isoallergens Pru p 1.0101, Pru p 1.0201 and Pru p 1.0301. In each plot, the black dot and dash line indicates the mean CSP plus one standard deviation. Residues whose ^{15}N chemical shifts were used for determining K_d are labeled.

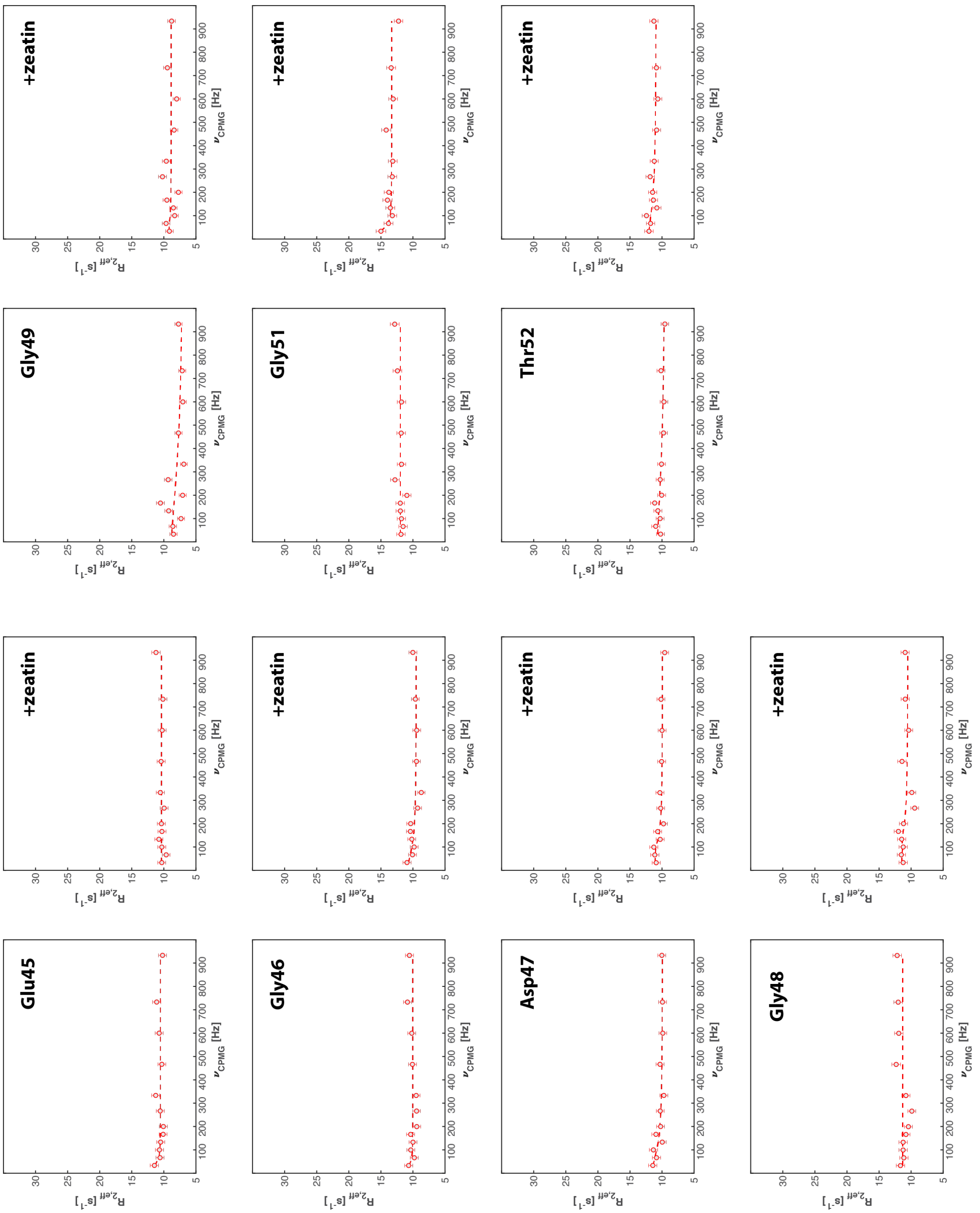

Supporting Figure 7. NMR relaxation dispersion (RD) data of Pru p 1.0101 before and after addition of zeatin (8-fold excess, ca. 50% saturation), recorded at 700 MHz. Backbone amide (^{15}N) RD profiles are shown for the glycine-rich region (Glu45-Thr52, with the exception of Pro50, which does not have a backbone amide NH for detection). All residues in this segment display near flat relaxation dispersion profiles with and without zeatin being bound, indicating that millisecond dynamics are absent.

	PDB entry code	Cavity Volume [a]	Cavity Volume [b]
LIPR-10.2B / chain A	2qim	663 $Å^3$	2050 $Å^3$
CSBP /chain A	2flh	309 $Å^3$	828 $Å^3$
Pru p 1.0101	6z98	534 $Å^3$	1291 $Å^3$

Supporting Table 1. Cavity volumes of zeatin binding PR-10 proteins, determined using [a] the program CASTp with the default probe radius of 1.4 Å and [b] the program 3V with inner and outer probe volumes of 1.3 Å and 3.0 Å, respectively.[1-2]

REFERENCES

1. Tian, W.; Chen, C.; Lei, X.; Zhao, J.; Liang, J., CASTp 3.0: computed atlas of surface topography of proteins. *Nucleic Acids Res.* **2018,** *46* (W1), W363-W367.
2. Voss, N. R.; Gerstein, M., 3V: cavity, channel and cleft volume calculator and extractor. *Nucleic Acids Res.* **2010,** *38* (Web Server issue), W555-W562.
